# The BH3-only protein NOXA serves as an independent predictor of breast cancer patient survival and defines susceptibility to microtubule targeting agents

**DOI:** 10.1038/s41419-021-04415-y

**Published:** 2021-12-13

**Authors:** Gerlinde Karbon, Manuel D. Haschka, Hubert Hackl, Claudia Soratroi, Lourdes Rocamora-Reverte, Walther Parson, Heidelinde Fiegl, Andreas Villunger

**Affiliations:** 1 Institute for Developmental Immunology, Biocenter, Medical University of Innsbruck, Innsbruck, Austria; 2 Insitute for Bioinformatics, Biocenter, Medical University of Innsbruck, Innsbruck, Austria; 3 Institute of Legal Medicine, Medical University Innsbruck, Innsbruck, Austria; 4 Department for Obstetrics & Gynecology, Medical University of Innsbruck, Innsbruck, Austria; 5 Present Address: Institute for Biomedical Aging Research, University of Innsbruck, Innsbruck, Austria

**Keywords:** Cancer, Translational research

## Abstract

Breast cancer (BC) treatment frequently involves microtubule-targeting agents (MTAs), such as paclitaxel, that arrest cells in mitosis. Sensitivity to MTAs is defined by a subset of pro- and anti-apoptotic BCL2 family proteins controlling mitochondrial apoptosis. Here, we aimed to determine their prognostic value in primary tumour samples from 92 BC patients. Our analysis identified high *NOXA/PMAIP* mRNA expression levels as an independent prognostic marker for improved relapse-free survival (RFS) and overall survival (OS) in multivariate analysis in BC patients, independent of their molecular subtype. Analysis of available TCGA datasets of 1060 BC patients confirmed our results and added a clear predictive value of *NOXA* mRNA levels for patients who received MTA-based therapy. In this TCGA cohort, 122 patients received MTA-treatment and high *NOXA* mRNA levels correlated with their progression-free interval (PFI) and OS. Our follow-up analyses in a panel of BC cell lines of different molecular subtypes identified NOXA protein expression as a key determinant of paclitaxel sensitivity in triple-negative breast cancer (TNBC) cells. Moreover, we noted highest additive effects between paclitaxel and chemical inhibition of BCLX, but not BCL2 or MCL1, documenting dependence of TNBC cells on BCLX for survival and paclitaxel sensitivity defined by NOXA expression levels.

## Introduction

Breast cancer (BC) is with 13.3% the most common type of cancer in women [1]. BC can be classified into three main subfamilies according to the presence or absence of the hormone receptors for oestrogen (ER) and progesterone (PR) and the human epidermal growth factor receptor 2 (HER2) status: Luminal A/B (about 40%, ER^+^/PR^+/-^/HER2^-^), HER2^+^ (10-15%, ER^-^/PR^-^/HER2^+^) and those negative for all these marker, referred to as TNBC (15-20%, ER^-^/PR^-^/HER2^-^). TNBC therapy involves aggressive chemotherapies due to lack of clear molecular targets [2, 3]. PARP1 inhibitors, Olaparib and Talazobarib, and the immune checkpoint inhibitor Atezolizumab, targeting PD-L1, in combination with the microtubule targeting agent (MTA) paclitaxel are novel treatment strategies used, reviewed by Lyons [4].

MTAs, like vincristine or paclitaxel, inhibit microtubule dynamics [5]. This eventually activates the spindle assembly checkpoint (SAC) and triggers mitotic (M)-arrest when applied in tissue culture, eventually leading to apoptosis [6]. Paclitaxel shows success in treating metastatic breast and ovarian cancer, as well as various leukaemias [5, 7]. Although MTAs are largely successful, resistance and neurotoxicity limit their broader application [8]. One way to evade mitotic cell death (MCD) is to overexpress anti-apoptotic BCL2 proteins [9]. In BC, MCL1, BCL2 and BCLX are often found amplified [10, 11], making them more resistant to different types of therapeutics [12, 13], including paclitaxel [14]. MCD is a desired outcome in cancer therapy, yet clinical efficacy also involves alternative anti-proliferative and pro-inflammatory effects [15]. Of note, tumour cells often manage to escape cell death in a process called “mitotic slippage”, the premature exit from mitosis, triggered by the gradual decay of cyclin B levels below a critical threshold, allowing cell survival [16, 17].

We and others recently demonstrated that the molecular mechanism underlying MCD depends on the activity of BH3-only proteins, most notably BIM and NOXA, and the degradation of anti-apoptotic MCL1 [18]. We could further demonstrate that NOXA protein mediates the degradation of MCL1 during extended M-arrest and that knockdown of NOXA leads to MCL1 stabilisation and resistance to MTAs in HeLa cervical cancer and A549 lung cancer cells [18]. In a follow-up study, we reported that the co-degradation of NOXA/MCL1 complexes during extended M-arrest requires the mitochondrial E3-ligase MARCH5 [19], suggesting that its inhibition may help increase the efficacy of MTAs. Interestingly, ablation of the mitochondrial GTPase DRP1, deregulating mitochondrial network dynamics, sensitizes epithelial cancer cells to MTA-induced apoptosis [20]. Taken together, this places mitochondria at the core of mitotic cell death regulation and MTA treatment success.

Along this line, BH3 mimetics, inhibiting anti-apoptotic BCL2 proteins, are confirmed to be sufficient to prime cancer cells to various chemotherapeutics, including paclitaxel [21]. BH3-mimetics bypass the need for upstream inducers of BH3-only proteins, such as p53 or PTEN, which are frequently impaired in human cancers. The first valid prototype of a BH3-mimetic, ABT-737, targeting BCL2, BCLX and BCLW, showed promising efficacy in haematological malignancies [22]. However, several cancer types are resistant to ABT-737, or its orally bioavailable successor, Navitoclax (ABT-263), due to the overexpression of MCL1 [23]. ABT-199 (Venetoclax), solely targeting BCL2 within the sub-nanomolar-range, shows clinical efficacy in CLL [24], is heavily explored in clinical trials and shows promising results in ER^+^ BC patient-derived xenotransplants (PDX) [25]. Wehi-539 and its successors, A-1155463 and A-1331852, are targeting BCLX within sub-nanomolar-range [26] but cause thrombocytopenia, limiting clinical application [27, 28]. More recently, a highly specific MCL1 inhibitor, S63845, was shown to have potent anti-tumour activity as a single-agent in preclinical leukaemia models [29], as well as in combination with ABT-199 in BC PDX studies [30].

Here, we tested the predictive value of BCL2 family expression levels for BC patient survival in a patient cohort with detailed clinical follow-up and investigated the biological significance of the NOXA/MCL1 axis for MCD in BC cell lines exposed to paclitaxel and BH3 mimetics.

## Results

### High *NOXA/PMAIP* mRNA expression predicts superior survival of BC patients receiving MTA-based therapy

**Fig. 1:**
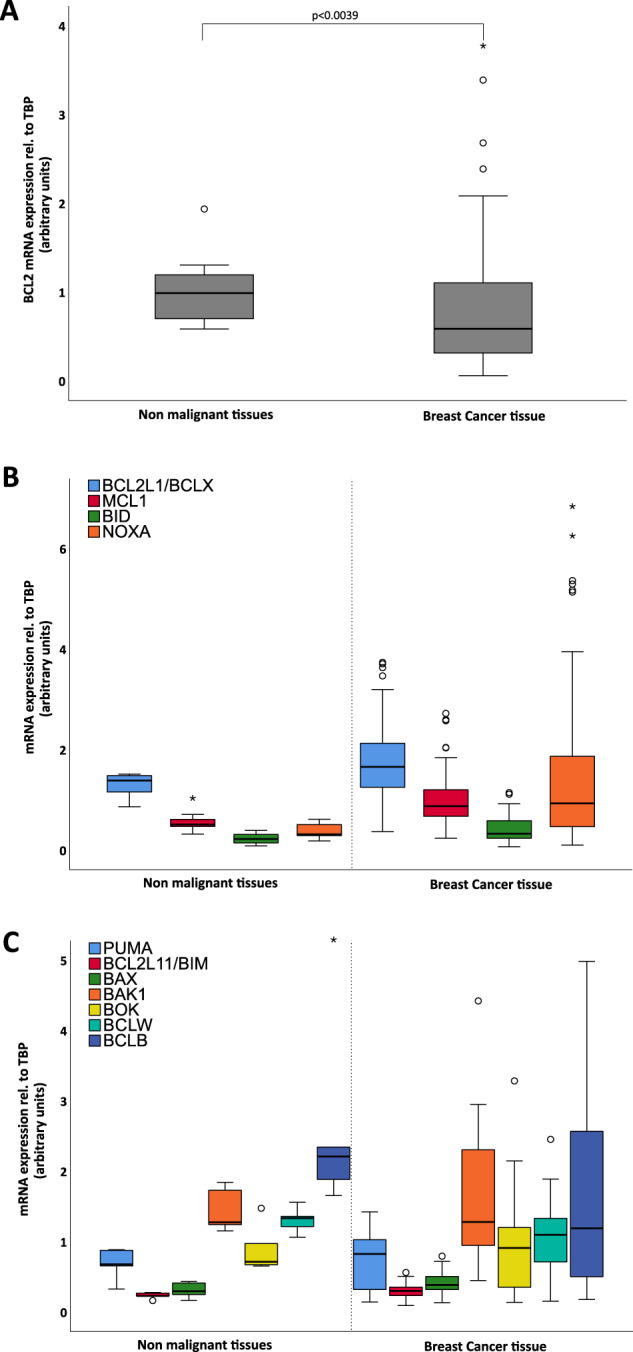
mRNA expression analysis of BCL2 family members in 10 non neoplastic and 92 neoplastic breast tissues. mRNA expression of **A** BCL2 (*p* = 0.039), **B** BCLX (*p* = 0.035), MCL1 (*p* = 0.001), BID (*p* = 0.020) and NOXA (*p* = 0.002) and **C** PUMA, BIM, BAX, BAK, BOK, BCLW and BCLB. Extreme values are marked with asterisks, outliers with circles.

We investigated the mRNA expression of pro-apoptotic effectors (BAX, BAK, BOK), BH3-only proteins previously involved in MTA-induced cell death (BID, BIM, PUMA, NOXA) and anti-apoptotic BCL2 family members (MCL1, BCL2, BCLX, BCLW, BCLB) within fresh-frozen tissue samples of different molecular subtypes from 92 patients with primary BC. Our first analysis revealed significant differences in relative mRNA levels between healthy and diseased tissue for anti-apoptotic *BCL2, BCLX/BCL2L1* and *MCL1*. Of note, *BCL2* expression was significantly lower in cancerous tissue (Fig. 1A), while *BCLX* and *MCL1* levels were found increased (Fig. 1B). Analysis of their pro-apoptotic counterparts, the BH3-only proteins *BID* and *NOXA*, revealed a significant increase in mRNA levels in cancerous compared to non-neoplastic tissue (Fig. 1B). In contrast, *PUMA* and *BIM/BCL2L11* levels and the effector proteins *BAX, BAK* or *BOK* or the anti-apoptotic proteins *BCLW* and *BCLB* were comparable (Fig. 1C). Pearson correlation analyses revealed significant associations for the co-expression of *MCL1* with *PUMA* or *BAX*, as well as *BCLW/BCL2L2* with *PUMA* or *BAX*. Amongst the pro-death proteins, an interdependence was noted between *BAX* and *BAK* or the BH3-only protein *PUMA* or *BID* with *BAX* (Suppl. Fig. 1A), suggesting co-regulation of gene expression.

When analysing clinical parameters, we confirmed previous findings [25] by documenting high *BCL2* mRNA expression in low-grade and luminal A type BC patients still expressing HR (Suppl. Table 1). Consistent with BCL2 being a target of HR signalling, levels were generally higher in HR^+^ tumours (Suppl. Table 1). BCLX expression correlated with the same parameters and was enriched in *HER2*^*+*^ tumours but no longer associated with low tumour grade. Neither *MCL1* nor *BCLW* mRNA showed any correlation with clinical parameters, but *BCLW* showed higher expression in large tumours. Amongst the pro-apoptotic genes, expression of *BID* associated with high tumour grade while *NOXA* was found higher expressed in medullary carcinomas (Suppl. Table 1).

**Fig. 2:**
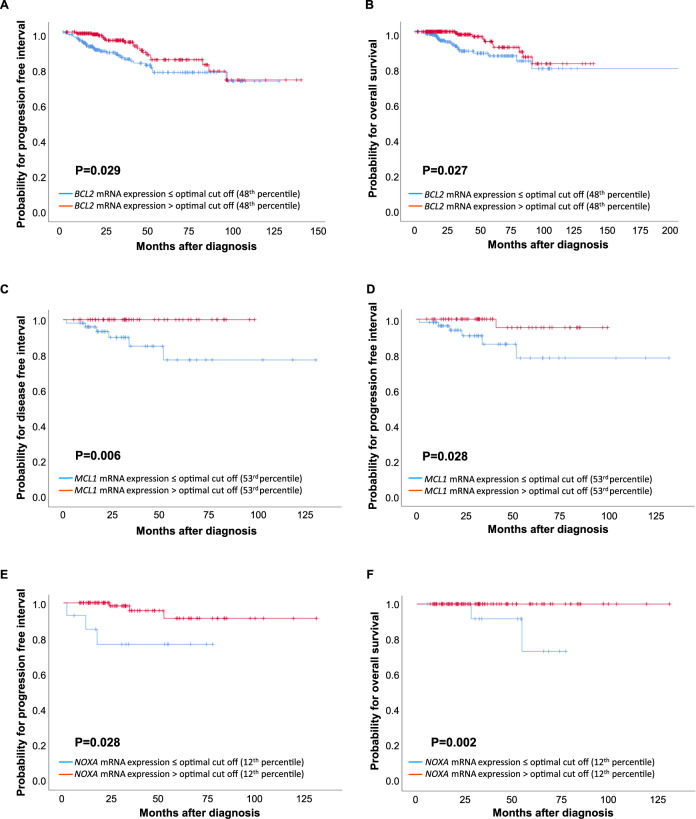
Kaplan–Meier survival analysis in the TCGA cohort. **A** PFI and **B** OS based on BCL2 mRNA-expression in 471 BC patients treated with chemotherapy other than MTA. **C** DFI and **D** PFI based on MCL1 mRNA-expression in 112 BC patients treated with MTA chemotherapy. **E** PFI and **F** OS based on NOXA mRNA-expression in 112 BC patients treated with MTA chemotherapy.

Performing ROC analyses to define the best cut-off to identify significant patient survival differences across cancer subtypes revealed a clear correlation between higher *BCL2* expression levels and a superior RFS. Higher levels of *MCL1* correlated significantly with a good OS in univariate analyses (Table 1). Of note, *NOXA* and *BOK* expression showed a strong correlation with both RFS and OS. While high *NOXA* expression was associated with superior survival, strong *BOK* expression was associated with poor survival (Table 1). Importantly, this correlation pattern for *BOK* and *NOXA* was maintained when multivariate analyses were performed (Table 2). Expression of *BCL2* still correlated with RFS, while levels of *MCL1* correlated only with improved OS (Table 2). Notably, these correlations were verified by analysing the TCGA database containing expression data of 1060 BC patients; from those, 471 patients were treated with chemotherapy, from which 112 patients received MTAs (Fig. 2A–D). Strikingly, *NOXA* mRNA expression levels correlated with OS and PFI within BC patients from the TCGA dataset treated with MTA but no other type of chemotherapy (Fig. 2E, F), showing its relevance and confirming the predictive value of our data (Table 2). Selected patient samples were also tested for NOXA protein levels, confirming a correlation between mRNA and protein levels (Suppl. Fig. 1B). Within the TCGA validation cohort, however, BOK expression levels no longer correlated with survival (data not shown).

**Table 1:** Univariate survival analysis of relapse free and overall survival of 92 patients with primary breast cancer diagnosed and treated at the Medical University of Innsbruck, AT.

Variable		Relapse-free survival		Overall survival	
		Median, years (95% CI)	*P*-value (logrank-test)	Median, years (95% CI)	*P*-value (logrank-test)
**Size**	T1	n.r.	0.150	n.r.	0.093
	T2/3/4	8.09 (0.00–19.35)		10.44 (4.62–16.25)	
**LN**	Negative	n.r.	0.112	n.r.	**0.007**
	Positive	n.r.		9.93 (6.35–13.50)	
**Tumour grade**	I	n.r.	0.609	10.73 (8.69–12.78)	0.886
	II	8.09 (n.r.)		14.64 (8.81–20.47)	
	III	n.r.		16.91 (8.76–20.06)	
**Histology**	invasive lobular breast cancer	n.r.	0.187	18.69 (5.86–31.53)	0.905
	invasive ductal breast cancer	8.09 (0.00–17.91)		12.15 (6.51–17.79)	
	medullary breast cancer	n.r.		16.91 (5.20–28.62)	
**MP**	premenopausal	8.09 (0.00–19.69)	0.492	n.r	0.106
	postmenopausal	n.r.		10.73 (5.33–16.14)	
**HER2**	neg	n.r.	0.127	16.91 (12.90–20.92)	0.067
	pos	5.23 (3.85–6.62)		7.25 (3.22–11.28)	
**ER**	neg	n.r.	0.646	16.91 (5.01–28.81)	0.696
	pos	19.23 (2.81–35.65)		14.64 (8.94–20.35)	
**PR**	neg	n.r.	0.709	10.20 (0.00–22.45)	0.376
	pos	19.23 (3.15–35.32)		14.64 (9.48–19.80)	
**MTA chemotherapy**	no	5.95 (0.00–16.78)	0.057	8.76 (6.60–10.92)	**0.001**
	yes	nr.		n.r.	
**Radiation therapy**	no	n.r.	0.316	14.64 (9.68–19.61)	0.870
	yes	6.77 (0.00–17.27)		17.78 (9.74–25.83)	
**Endocrine therapy**	no	n.r.	0.658	14.64 (4.38–24.90)	0.601
	yes	8.09 (0.00–18.43)		12.15 (6.23–18.07)	
***BCL2*** **mRNA expression**	low (≤48^th^ percentile)	4.72 (2.07–7.36)	**0.003**	8.59 (2.04–15.14)	0.117
	high (>48^th^ percentile)	n.r.		15.46 (7.41–23.52)	
***MCL1*** **mRNA expression**	low (≤53^rd^ percentile)	5.95 (n.r.)	0.128	9.35 (7.11–11.59)	**0.014**
	high (>53^rd^ percentile)	19.23 (n.r.)		17.95 (n.r.)	
***NOXA*** **mRNA expression**	low (≤12^th^ percentile)	1.98 (1.44–2.52)	**<0.001**	3.28 (1.64–4.92)	**<0.001**
	high (>12^th^ percentile)	n.r.		16.91 (13.39–20.43)	
***BOK*** **mRNA expression**	low (≤34^th^ percentile)	n.r.	**0.008**	n.r.	**0.010**
	high (>34^th^ percentile)	5.95 (2.70–9.20)		10.44 (6.92–13.96)	

*Note:* The significance level (*P*) was determined by log-rank test.

*LN* lymph node status, *MP* menopausal status, *HER2* human epidermal growth factor receptor 2 status, *ER* oestrogen receptor status, *PR* progesterone receptor status, *HR* hormone receptor status, *n.r.* not reached.

*p* values that are statistically significant are shown in bold.

**Table 2:** Multivariate Cox-regression analysis of relapse free survival and overall survival of 92 patients with primary breast cancer diagnosed and treated at the Medical University of Innsbruck, AT.

		Relapse-free survival		Overall survival	
Variable		HR of relapse (95% CI)	*P*-value	HR of death (95% CI)	*P*-value
**Size**	T1 vs. T2/3/4	1.63 (0.66–4.02)	0.289	1.49 (0.60–3.69)	0.386
**LN**	neg. vs. pos.	1.79 (0.75–4.29)	0.193	2.91 (1.17–7.24)	**0.022**
**Tumour grade**	grade I vs. grade II/III	1.94 (0.42–8.90)	0.394	1.02 (0.34–3.10)	0.975
**MP**	pre vs. post	0.63 (0.34–1.19)	0.157	1.35 (0.70–2.62)	0.369
**HR**	neg. vs. pos.	0.87 (0.42–1.80)	0.709	0.80 (0.42–1.54)	0.507
**HER2**	neg. vs. pos.	1.47 (0.74–2.92)	0.269	1.76 (0.91–3.40)	0.091
**MTA chemotherapy**	no vs. yes	2.00 (0.95–4.21)	0.067	3.31 (1.35–8.16)	**0.009**
***BCL2*** **mRNA expression**	Low vs. high (<or>48^th^ percentile)	0.31 (0.15–0.66)	**0.002**	—	—
***MCL1*** **mRNA expression**	Low vs. high (<or>53^rd^ percentile)	—	—	0.41 (0.22–0.79)	**0.008**
***NOXA*** **mRNA expression**	Low vs. high (<or>12^th^ percentile)	0.14 (0.06–0.32)	**<0.001**	0.15 (0.06–0.38)	**<0.001**
***BOK*** **mRNA expression**	Low vs. high (<or>34^th^ percentile)	4.16 (1.74–9.93)	**0.001**	4.47 (2.05–9.78)	**<0.001**

*Note*: The significance level was determined by Cox regression analysis.

*HR* hazard ratio, *LN* lymph node status, *MP* menopausal status, *HER2* human epidermal growth factor receptor 2 status, *RR* relative risk.

*p* values that are statistically significant are shown in bold.

### NOXA protein expression defines MTA-sensitivity in TNBC cell lines

We further investigated the expression profile of NOXA and its relevance for MTA-treatment in relation to other members of the BCL2 protein family in more detail. Therefore, we chose eight different BC cell lines, representing the three main subfamilies: Luminal A/B (MCF-7, T47D and ZR-75-1), TNBC (HS-578-T, MDA-MB-231, Cal-51 and BT20) and HER2^+^ (SKBR3) and analysed protein expression levels of the most common pro-and anti-apoptotic proteins.

**Fig. 3:**
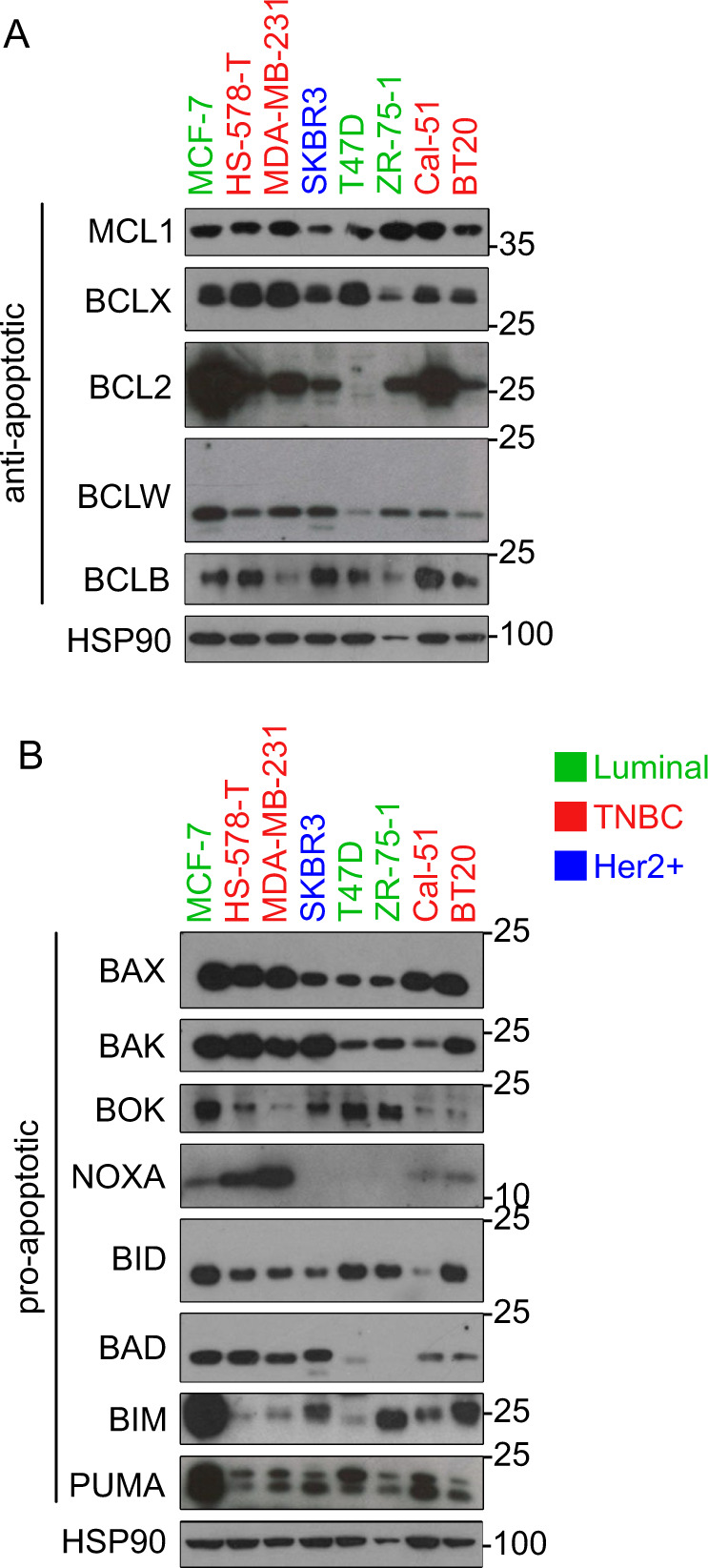
BCL2 protein family expression in different BC cell lines. Different BC cell lines were subjected to western blotting using the indicated antibodies recognising **A** anti- and **B** pro-apoptotic BCL-2 proteins. HSP90 was used as a loading control.

The expression of BCL2, NOXA, BIM, BCLB and BOK differed substantially amongst cell lines, whereas MCL1, BCLX, or BID expression levels show less variability (Fig. 3A, B). The protein expression of NOXA was highest in the TNBC cell lines, while BOK was hardly detectable in this subset. MDA-MB-231, HS-578-T and T47D also showed a slightly higher expression of BCLX than the other BC cell lines (Fig. 3A), suggesting co-dependence on BCLX for survival. The strong expression of different BCL2 pro-survival proteins suggests variable dependency for cell survival that does not correlate with a particular molecular subtype, which we investigated in the next step using selective BH3 mimetics.

### BH3-mimetics efficiently enhance the effects of paclitaxel

**Fig. 4:**
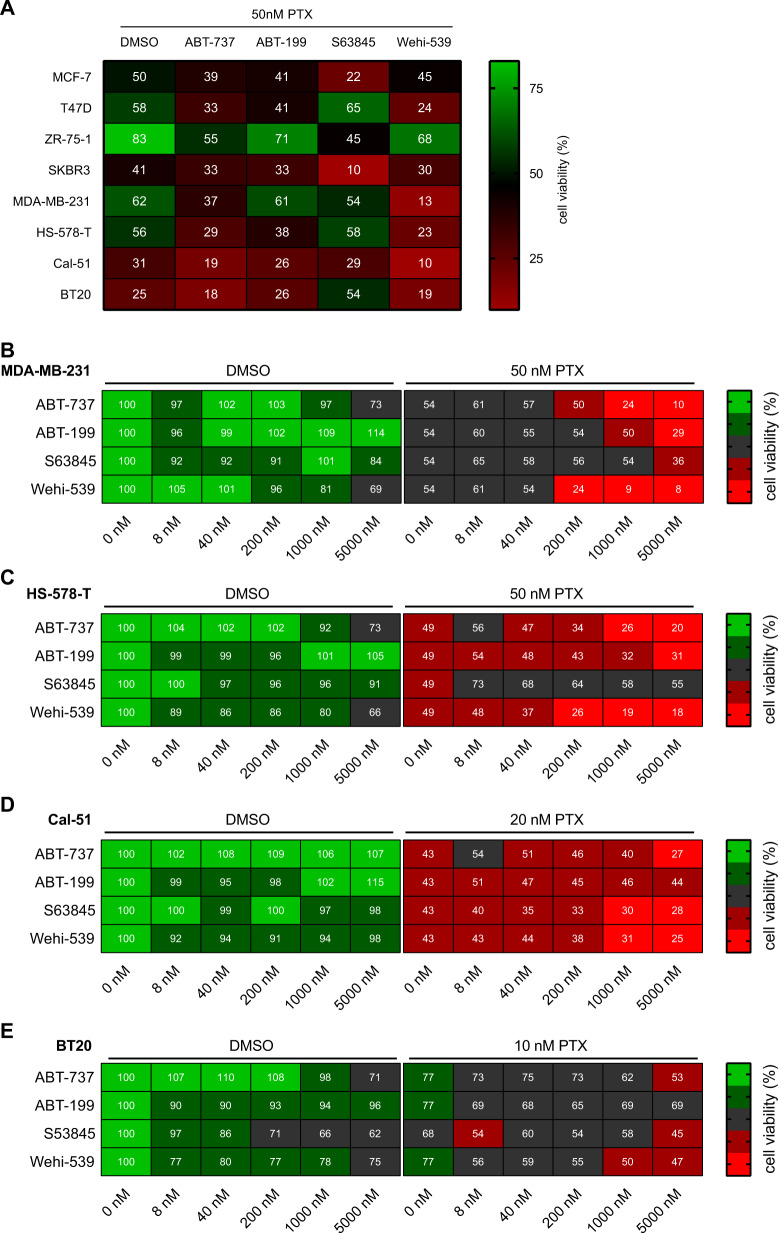
Paclitaxel increases the sensitivity towards BH3 mimetics. **A** The indicated cell lines were treated with 50 nM paclitaxel alone or in combination with 1 µM of BH3mimetics for 48 h. **B**–**E** TNBC cell lines were treated with graded concentrations of BH3-mimetics in combination with a predetermined fixed concentration of paclitaxel for 48 h. Metabolic activity was calculated by setting the metabolic activity in relation to the DMSO control. Heatmaps show mean values of metabolic activity ranging from 0 to 100% as assessed by MTT-assay. **A** All cell lines (*n* = 3–4), **B** MDA-MB-231 (*n* = 6), **C** HS-578-T (*n* = 4), **D** Cal-51 (*n* = 6), **E** BT20 (*n* = 6).

All cell lines were treated either with fixed concentrations of paclitaxel alone or in combination with a graded concentration of the different BH3 mimetics, including ABT-737, ABT-199, S63845 and Wehi-539 (Suppl. Fig. 2), or vice versa (Suppl. Fig. 3). MTT-assay was used as an indirect readout for cell viability. As expected, the cell lines showed different sensitivity against paclitaxel which did not correlate with a particular molecular subtype. Two of the TNBC cell lines, Cal-51 and BT20, were most sensitive to MTA-treatment. Their metabolic activity dropped to about 31% and 25%, respectively, when treated with paclitaxel alone (Fig. 4A). In contrast, the ER^+^ cell lines ZR-75-1 (83%), T47D (58%) and MCF-7 (50%) showed reduced MTA-sensitivity (Fig. 4A). A saturation using 50 nM paclitaxel was visible for all cell lines tested. Higher concentrations did not reduce metabolic activity any further, with the notable exception of the HS-578-T cells (Suppl. Fig. 3).

Inhibiting the pro-survival BCL2 proteins with the different BH3 mimetics alone was mostly ineffective. The BCL2 inhibitor ABT-199 did not affect any cell line at the assayed concentrations (up to 5 µM), including MCF7 cells, which showed the highest BCL2 expression. Similar, the MCL1 inhibitor S63845 only affected MCF-7 and SKBR3 cell lines (Suppl. Fig. 2). However, ABT-737, inhibiting BCL2, BCLX and BCLW, and the BCLX inhibitor, Wehi-539, showed activity in some cell lines when used at high doses as a single agent (Fig. 4B–E). Notably, combining paclitaxel with BH3 mimetics led to additive effects, despite variations in their overall potency (Fig. 4, Suppl. Figs. 2, 4, 5). In combination with the MCL1 inhibitor S63845, paclitaxel most potently reduced the metabolic activity of MCF7 cells from 50% (paclitaxel) to 22% (paclitaxel + S63845; Fig. 4A). The combination of paclitaxel with the BCL2 inhibitor ABT-199 had only a mild effect on HS-578-T or MCF-7 cells despite their high BCL2 levels. (Fig. 4A, Suppl. Figs. 2, 4, 5).

Potent effects on all cell lines tested were consistently seen when paclitaxel was combined with ABT-737, having the most substantial impact on the TNBC cell lines HS-578-T and MDA-MB-231. Their metabolic activity was further reduced on average by 27% and 25%, respectively, in the combination setting (Fig. 4A). This finding points towards a critical role for BCLX for cell survival after paclitaxel treatment. Consistently, the most pronounced effects were seen using paclitaxel together with the selective BCLX inhibitor, Wehi-539. This inhibitor further decreased metabolic activity by 33% and 49% in the HS-578-T and MDA-MB-231, respectively (Fig. 4). An increase of the paclitaxel concentration to 500 nM combined with Wehi-539 eliminated all viable MDA-MB-231 cells (Suppl. Fig. 3A).

To optimise the effect between paclitaxel and BH3 mimetics, we chose the estimated LD50 concentration of paclitaxel and titrated the different BH3 mimetics. Only inhibition of BCLX, by using ABT-737 or Wehi-539, showed effects as single agents at the highest drug concentration used, i.e., 5 µM. (Fig. 4B–E, Suppl. Figs. 2, 4, 5). An additive effect was best seen in the TNBC cell lines, identifying BCLX as their primary survival factor that, together with the NOXA/MCL1 axis, may define responsiveness to MTAs.

### The NOXA/MCL1 axis controls paclitaxel-induced cell death in TNBC cell lines

We could previously show that NOXA driven degradation of MCL1 during extended M-arrest promotes cell death [18]. This may explain why chemical inhibition of MCL1 had little effect on paclitaxel sensitivity, as it is automatically degraded upon paclitaxel treatment. We chose the TNBC cell lines HS-578-T, MDA-MB-231 and Cal-51 to analyse the relevance of the NOXA/MCL1 axis for MCD as they showed the strongest NOXA expression. Of note, while MCL1 levels were comparable in these cell lines, NOXA expression appears graded, with the MDA-MB-231 cell line having the highest, followed by HS-578-T and the Cal-51 showing the lowest NOXA levels (Fig. 3).

**Fig. 5:**
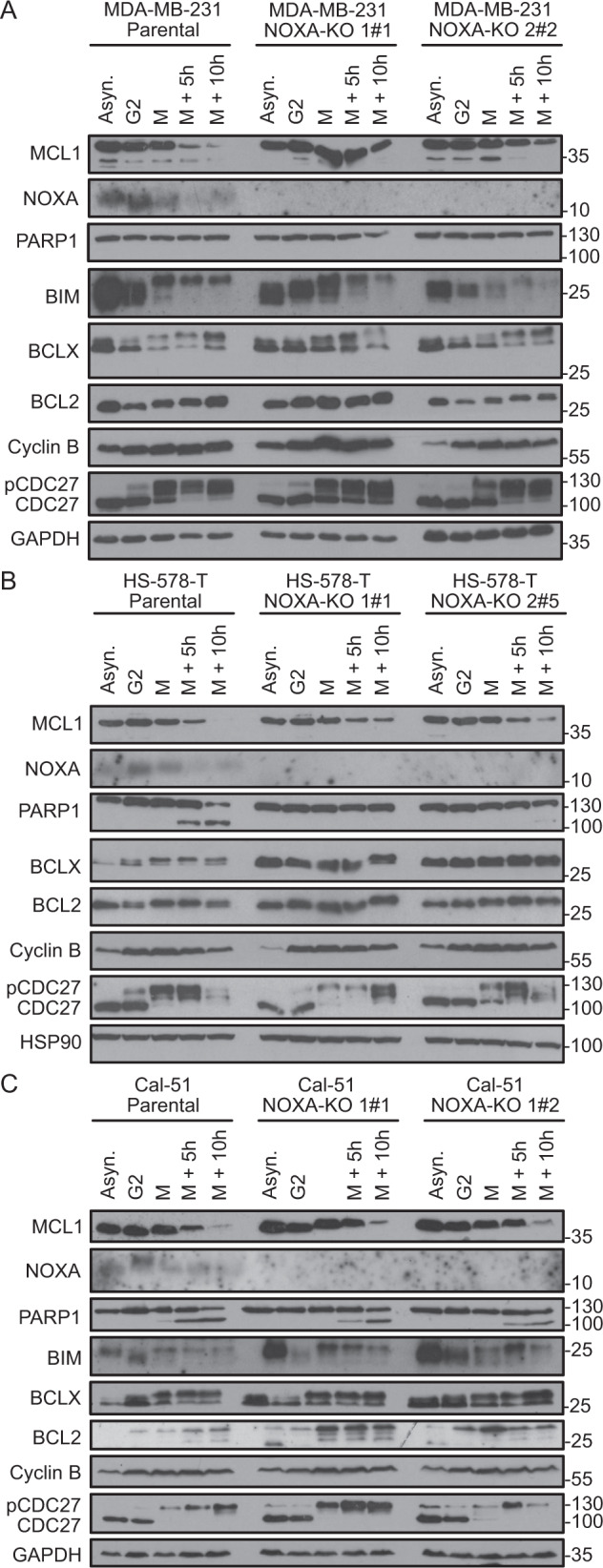
TNBC cell lines treated with paclitaxel show MCL1 and NOXA co-degradation. **A**–**C** TNBC proficient (Parental) or deficient in NOXA (NOXA-KO) were left asynchronous (Asyn.) or synchronised with a double-thymidine block and released into 500 nM PTX. Cells were harvested in G2 and early M-phase (M); part of the M-phase cells was reseeded and harvested 5 h (M + 5 h) and 10 h (M + 10 h) later. Harvested cells were subjected to western analysis using the indicated antibodies.

All three cell lines were synchronised with a double-thymidine arrest and released into paclitaxel-containing media to induce prolonged M-arrest. Mitotic shake off was used to enrich cells arrested in mitosis. CDK1-mediated phosphorylation of CDC27 in mitosis, a component of the anaphase-promoting complex (APC), validated the synchronisation procedure (Fig. 5). We observed that MCL1 and NOXA are co-degraded during extended M-arrest in all three TNBC cell lines. Overall, the NOXA levels followed MCL1 expression and peaked in G2/M before being co-degraded (Fig. 5)

The reduction of MCL1/NOXA levels correlated well with apoptosis onset, as monitored by caspase-mediated cleavage of PARP1. Cal-51 cells showed the strongest PARP1 cleavage during extended M-arrest, followed by HS-578-T cells. In strong contrast, MDA-MB-231 cells showed no detectable PARP1 cleavage, indicating resistance against paclitaxel (Fig. 5A). These patterns fit the observed paclitaxel sensitivity/resistance phenotypes noted in the MTT-assays presented above (Fig. 4A, Suppl. Fig. 3F).

All three cell lines showed the described phosphorylation of BIM in mitosis, which promotes its APC^CDC20^-driven degradation [18, 31]. BCLX and BCL2 are well known to be phosphorylated during mitosis [9, 32–34]; this was best observed for BCLX, most of it migrating significantly slower in SDS-PAGE, less notable for BCL2, at least with the antibody used (Fig. 5).

To assess the relevance of NOXA/MCL1 turnover for tumour cell survival, we generated NOXA-KO derivatives from these three TNBC cell lines using two independent *sgRNAs* targeting *NOXA*. While the steady-state levels of MCL1 did not substantially differ in asynchronous cells, we could observe a clear stabilisation of MCL1 in the absence of NOXA compared to parental cells upon extended M-arrest (Fig. 5). HS-578-T cells showed the most robust MCL1 stabilisation upon M-arrest, followed by the MDA-MB-231 cells, while this effect was modest in the Cal-51 cell line. Regardless, the absence of NOXA was beneficial for survival upon MTA-treatment, as PARP1 cleavage was strongly reduced in the HS-578-T (Fig. 5B) and the Cal-51 cells (Fig. 5C). As there was no PARP1 cleavage detectable in the parental MDA-MB-231 cells, no such effect was observed in NOXA-KO cell lines (Fig. 5A).

### NOXA promotes paclitaxel-induced cell death and synergy with BH3-mimetics

**Fig. 6:**
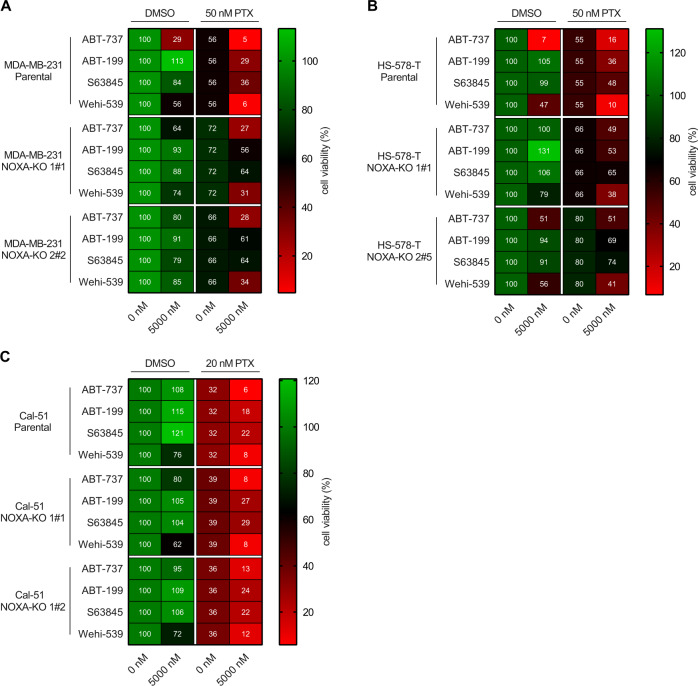
NOXA deletion protects TNBC from PTX and BH3-mimetic co-treatment. Parental and two NOXA-KO clones of each TNBC cell line were treated with or without 5000 nM of the indicated BH3-mimetics alone (DMSO) or in combination with 20 or 50 nM PTX for 48 h. Heatmaps are showing the mean value of metabolic activity assessed with MTT-assay. **A** MDA-MB-231 (*n* = 5), **B** HS-578-T (*n* = 4), **C** Cal-51 (*n* = 5).

Monitoring PARP1 cleavage by western blot may not have been sensitive enough to reveal a contribution of NOXA to paclitaxel-induced cell death, either when used alone or in combination with BH3-mimetics. Hence, to assess if NOXA-deficiency provides MDA-MB-231 cells with a potential survival benefit, we treated these cells with paclitaxel plus BH3-mimetics (Fig. 6, Suppl. Fig. 5). The metabolic activity was reduced in the parental cell line by paclitaxel to a mean of 56%, remaining higher in the NOXA-KO clones (72% and 66%, respectively). This effect became more prominent when paclitaxel was combined with different BH3-mimetics (Fig. 6A, Suppl. Fig. 4A, Suppl. Fig. 5). Again, ABT-737 and Wehi-539 showed the most potent effects in the parental cell line, indicative of their BCLX dependence in the presence of MTAs. Accordingly, viability was partially rescued by the deletion of NOXA. Synergy between PTX and Wehi-539 and NOXA-dependence were also detectable by western blotting in unsynchronised cells using PARP1 cleavage as a readout. Again, MCL1/NOXA complexes were degraded over time in PTX treated cells and loss of NOXA reduced PARP1 cleavage. Expression levels of BCLX, BCL2, BID or BIM did not change substantially but showed mobility shifts consistent with phosphorylation in mitosis (Suppl. Fig. 6). Similar results were seen in the HS-578-T cell lines upon *NOXA* deletion (Fig. 6B, Suppl. Fig. 5). In the Cal-51 cells, the deletion of *NOXA* conveyed only modest paclitaxel resistance when compared to the other two TNBC cell lines, consistent with the moderate impact on PARP1 cleavage (Fig. 5C).

## Discussion

Analysing a well-characterised set of 92 BC patient specimens that have subsequently been treated with chemotherapy post-surgery, *NOXA* was identified as the only BH3-only protein with prognostic value across all molecular BC subtypes. High mRNA expression strongly correlated with improved RFS and OS within uni- and multivariate Cox-Regression analysis (Tables 1, 2). This observation could be confirmed in an independent BC patient cohort of the TCGA dataset. Most notably, *NOXA* levels also predicted superior PFI and OS in the 122 TCGA patients treated with MTAs, but not for the 350 patients receiving other chemotherapeutic agents (Fig. 2E, F).

Of note, mRNA levels of BOK, implicated in endoplasmic reticulum stress induced apoptosis [35], also showed a highly significant prognostic value in uni- and multivariate analysis (Tables 1, 2), but not the TCGA data set. BOK has been implicated in Ca^++^ signalling from the ER [36] and pyrimidine synthesis thereby affecting drug resistance and cell proliferation rates [37]. Yet, it remains unclear how low BOK expression would benefit BC patients. A more detailed analysis of this protein in BC appears warranted, in particular as loss of BOK reportedly prevents liver cancer in mice [38].

Looking at the predictive value of anti-apoptotic BCL2 proteins, we confirmed high mRNA levels of *BCL2* as beneficial for RFS, as noted before [39, 40], and reconfirmed this within the TCGA dataset (Fig. 2A, B). A similar beneficial effect could be linked to *MCL1* mRNA expression (Fig. 2C, D). It remains a matter of debate why high levels of a pro-survival protein may improve BC patients’ prognosis. Still, one can imagine a scenario where high BCL2 or MCL1 expression may reduce the pressure to delete other cell death regulators, such as p53, which comes at the price of impaired genomic stability [41]. In fact, BCL2 overexpression has been shown to delay tumour onset in animal models of irradiation-driven lymphoma and myelodysplastic syndrome transition into AML [42, 43]. MCL1 is a short-lived protein regulated mainly at the translational and post-translation level; hence, analysis of protein levels is critical. Consistently, studying a panel of tumour tissue specimens on a tissue microarray revealed that high expression of MCL1 predicts poor outcome in BC in all but the HER2 amplified subtype [44]. Moreover, MDA-MB-468 TNBC were highly susceptible to chemical MCL1 inhibition or genetic ablation and tumours forming in the MMTV-PyMT mouse model of BC showed clear MCL1 dependence [44]. Consistent with these observations, a recent study reported the beneficial effects of chemical MCL1 inhibition and the MTA docetaxel in TNBC patient-derived xenotransplant (PDX) studies in mice [30].

Our characterisation of the BCL2 protein family expression in cell lines revealed high variation across subtypes (Fig. 3). The low expression of NOXA in SKBR3, T47D and ZR-57-1 (Fig. 3B) might indicate its downregulation as part of a selected survival mechanism. Resistance to therapy is frequently correlated with the downregulation of *NOXA* mRNA in multiple cancer types [45] and linked to the fact that NOXA plays a decisive role in the degradation of MCL1 [18, 46].

The TNBC MDA-MB-231 cell line showed an above-average resistance to paclitaxel, compared to the two other TNBC cell lines, which may be related to an increased propensity to undergo mitotic slippage [47, 48]. Low BAK levels were shown to increase resistance against paclitaxel [49], consistent with MCL1 preferentially binding to BAK [46]. We could observe this for the ZR-75-1 and T47D cell line (Fig. 3B, Suppl. Fig. 3F, G). However, Cal-51 cells also showed a low BAK expression but are among the most paclitaxel-sensitive cell lines (Fig. 3B, Suppl. Fig. 3C), suggesting that BAK expression levels alone cannot be seen as a reliable predictor of paclitaxel sensitivity.

Similarly, the expression of BCL2 proteins among our BC cell lines did not allow predictions on their sensitivity against BH3-mimetics, best seen in MCF-7 cells when comparing BCL2 expression with sensitivity to ABT-199. Still, those cells were susceptible to inhibition of MCL1. This indicates that, despite frequent BCL2 upregulation, BCL2 is not a major survival protein for these tumour cells. However, an earlier study using ER^+^ PDX models could show that treating luminal BC with ABT-199 was as effective as ABT-737 combined with chemotherapy [25]. This suggests that BCL2 can become a critical survival factor when its co-expressed pro-survival partners are neutralised.

In a genome-wide siRNA screen, MCL1 was shown to be a critical survival factor in TNBC cells [50]. We used one of the latest MCL1 inhibitors in clinical development, S63845 [29]. However, it showed limited potency against TNBC cell lines when used as a single agent. Yet, the HER2^+^ SKBR3 were highly sensitive to S63845 alone, which correlates with the finding that these cells rely on MCL1 for survival [51]. While combining MCL1 inhibition with paclitaxel may be beneficial in some settings [30], our data show that BCLX seems to be generally more critical than MCL1 for BC survival (Fig. 4, Suppl. Fig. 2), in line with earlier observations [52]. BH3-profiling in MDA-MB-231 cells revealed its dependency on BCLX to antagonise pro-apoptotic function [53]. However, as a single agent, Wehi-539 was shown to have only a minor effect on the metabolic activity of TNBC, including the MDA-MB-231 [54], fitting our data. Nonetheless, combining Wehi-539 with MTAs in TNBC cell lines reveals a BCLX dependence (Fig. 5), suggesting higher therapy efficacy [55], probably triggered by IFN/IRF3-driven upregulation of NOXA [56]. P53 regulated expression of NOXA seems to play a minor role in this setting, as MDA-MB231 and HS-578-T cells express only mutant p53. In line with these results, elevated levels of BCL2 and MCL1 can lead to resistance against Wehi-539 [26]. An improved version of Wehi-539, with oral activity, might allow the use of lower doses to avoid thrombocytopenia while still maintaining its anti-cancer efficacy [28]. In vivo studies of the BCLX inhibitor, A-1331852, already showed an enhancement of the effectiveness of paclitaxel [57], and new PROTAC-based concepts may facilitate clinical application avoiding thrombocytopenia [58, 59].

We could show that deleting NOXA leads to stabilisation of MCL1, giving TNBC a survival benefit during extended M-arrest (Fig. 5), in line with our previous studies in HeLa and A549 cells [18]. Stabilised MCL1 can bind BAK and/or sequester BIM, which otherwise would be free to activate the intrinsic apoptotic pathway [46, 60]. Upon NOXA dependent degradation of MCL1, more BIM is released from MCL1 sequestration and can execute apoptosis; this can be best seen in Cal-51 NOXA-KO cells (Fig. 5C), which show less protection against M-arrest when compared to MDA-MB-231 NOXA-KO cells. This might rely on the low NOXA expression per se, indicating that the NOXA/MCL1 axis might not be that prominent in this cell line or that these cells escape M-arrest by slippage [61]. The HS-578-T and MDA-MB-231 cell lines both showed a survival benefit upon NOXA deletion, as evident by the reduced PARP1 cleavage in the NOXA-KO cells (Fig. 6, Suppl. Fig. 6).

In summary, the NOXA/MCL1 axis plays a crucial role in TNBC treated with MTAs. Therefore, it could be helpful to increase or restore NOXA expression by inhibiting its degradation, e.g. by targeting the E3 ligase function of MARCH5 (19). This would allow a dosage reduction of both MTAs and BCLX inhibitors, thereby avoiding their respective side effects of neurotoxicity and thrombocytopenia [58, 59].

## Materials and methods

### Study design, patients and specimens

Expression levels were quantified by reverse-transcription PCR in mRNA isolated from freshly frozen tumour and adjacent tissue from 92 patients with primary BC treated with chemotherapy after surgery (aged 30.3 to 86.7; median age at diagnosis, 53.0 years) and 10 patients with benign breast diseases (aged 19.8 to 46.0; median age at diagnosis, 35.5 years) treated at the Department of Obstetrics and Gynaecology, Medical University of Innsbruck, Austria. Written informed consent is not available from all patients (specimen collections before the year 2000). But in accordance with the Austrian law, the study was reviewed and approved by the Ethics committee of the Medical University of Innsbruck (reference number: 1021/2017), it was conducted in accordance with the Declaration of Helsinki and in concordance with the Reporting Recommendations for Tumour Marker Prognostic Studies of the National Cancer Institute (REMARK) [62]. HR status was identified by immunohistochemistry (IHC). All samples were anonymized before analysis was performed, to guarantee the protection of privacy.

A power calculation for survival analysis was performed for BCL2 expression based on the univariate hazard ratio for mortality described by Dawson et al. (doi: 10.1038/sj.bjc.6605736) using the sample-size formula for the proportional-hazards regression model. The calculated, required total number of events was 16 (in our cohort 46 patients died, 41 had a relapse).

We analysed the BC dataset from The Cancer Genome Atlas (TCGA) project (*n* = 1060) described in [63, 64] including OS, DSS data and gene expression data from 471 resected primary breast tumours from patients treated with chemotherapy (aged 26.0 to 84.0 years; median age at diagnosis, 53.0 years).

### RNA isolation and reverse transcription for qRT-PCR

Total cellular RNA extraction, reverse transcription and PCR reactions were performed as previously described [65]. Primers and probe for the TATA box-binding protein (TBP; endogenous RNA-control) were used according to Bieche et al. [66]. Primerlist can be found in the Suppl. Material Table 3.

### Tissue culture and generation of NOXA KO lines

Cells were cultured in a humidified atmosphere containing 5% CO_2_ at 37 °C with the required media (Suppl. Material Table 1) and routinely checked for mycoplasma. Amplification of 15 *STR* and *amelogenin* loci was carried out in the Institute of Legal Medicine, Innsbruck Medical University [67] for fingerprinting the cell lines in use. Synchronisation with double-thymidine-arrest and generation of NOXA-KO (with CRISPR/Cas9 system) cells was performed as described previously [19]; guide sequences are found in Suppl. Material Table 3.

### Metabolic activity assessment

Metabolic activity was determined using the 3-(4,5-dimethylthiazol-2-yl)-2,5-diphenyltetrazolium bromide (MTT)-assay (Cell Proliferation Kit I, Roche Germany, Mannheim) according to manufacturer´s protocol. Cells were seeded in duplicates in 96-well flat-bottom plates and treated 24 h later with paclitaxel and/or graded doses of BH3-mimetics. DMSO was used as solvent control. Absorbance was measured at 570 nm and 650 nm using an ELISA plate reader (Sunrise, TECAN) with Magellan software (TECAN, V6.4). Metabolic activity was calculated in Excel, setting the OD-read of DMSO only treated cells to 100% metabolic activity.

### Cell lysis and immunoblot analysis

Cells lysis and immunoblot was performed as previously described [19], used antibodies listed in Suppl. Material Table 2.

### Statistical analysis

Mann–Whitney U test was applied to compare mRNA expression levels between groups. Univariate Kaplan-Meier analyses and multivariate Cox survival analyses were used to explore the association of *BCL2* family members mRNA expression levels with survival. Optimal thresholds for survival analyses were identified using Youden’s index [68] based on a receiver operating characteristic (ROC) curve analysis; analyses were performed using SPSS statistical software (version 26.0; SPSS Inc.).

One-tailed paired Student *t*-test was calculated on Prism8 (GraphPad Software) for Suppl. Fig. 3. Data in Suppl. Fig. 3 are represented with standard errors of the mean (SEM). Statistical significance is shown with symbols: *p-value < 0.05, **p-value < 0.01 and ***p-value < 0.001.

## Supplementary information

Suppl. Figs. 1-6

Suppl. Table 1

Suppl. legends

Suppl. materials

AJ checklist

## Data Availability

The datasets generated during and/or analysed during the current study are available from the corresponding author on reasonable request.
