# Supplementary material for: The BH3-only protein NOXA serves as an independent predictor of breast cancer patient survival and defines susceptibility to microtubule targeting agents
Source: Cell Death Dis. 2021 Dec 13;12(12):1151. doi: 10.1038/s41419-021-04415-y (PMC8668920; doi:10.1038/s41419-021-04415-y)

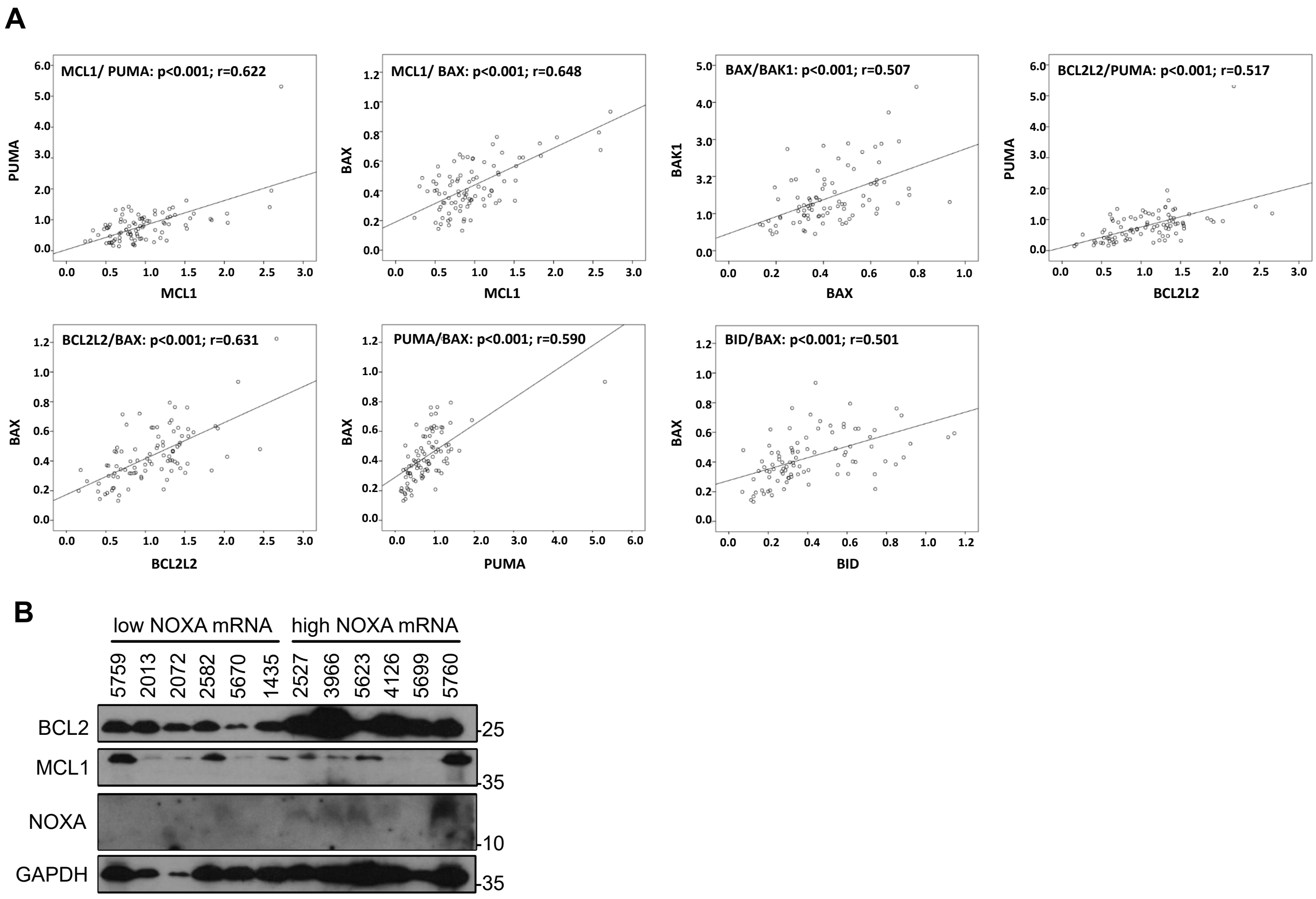
A
MCL1/ PUMA: p<0.001; r=0.622
MCL1/ BAX: p<0.001; r=0.648
BAX/BAK1: p<0.001; r=0.507
BCL2L2/PUMA: p<0.001; r=0.517
BCL2L2/BAX: p<0.001; r=0.631
PUMA/BAX: p<0.001; r=0.590
BID/BAX: p<0.001; r=0.501
PUMA
BAX
BAK1
MCL1
BCL2L2
BID
B
low NOXA mRNA
high NOXA mRNA
5759 2013 2072 2582 5670 1435 2527 3966 5623 4126 5699 5760
BCL2
MCL1
NOXA
GAPDH
25
35
10
35

Supp Fig 2

Gerlinde Karbon et al

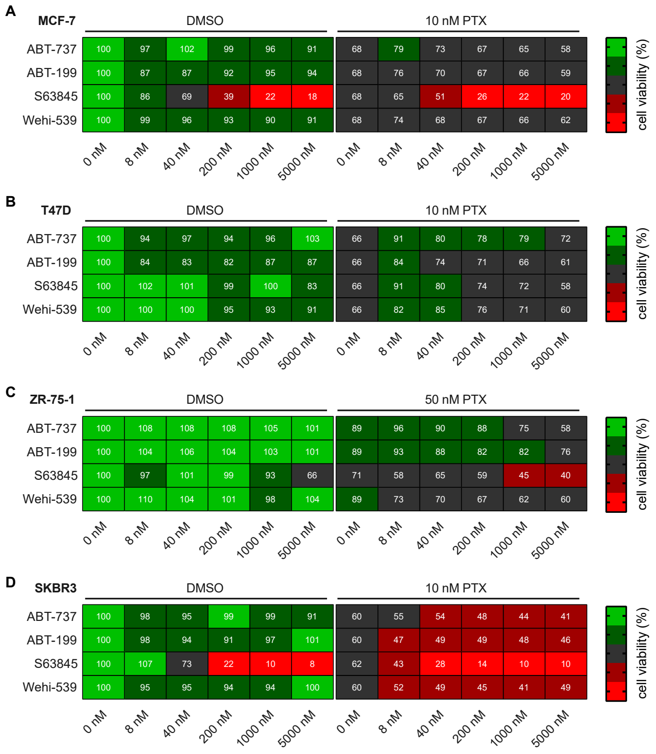

Supp. Figure 3

Gerlinde Karbon et al

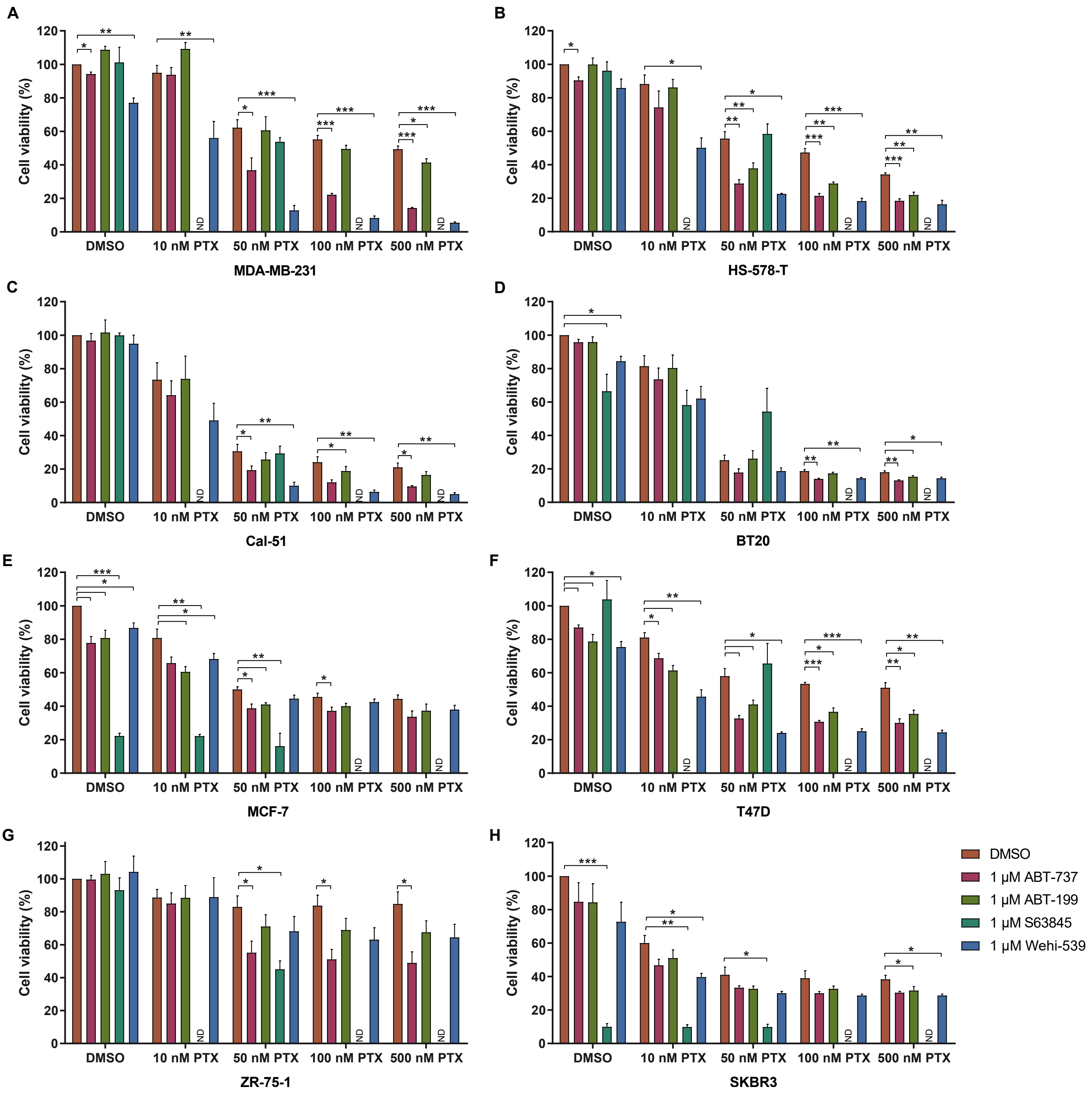

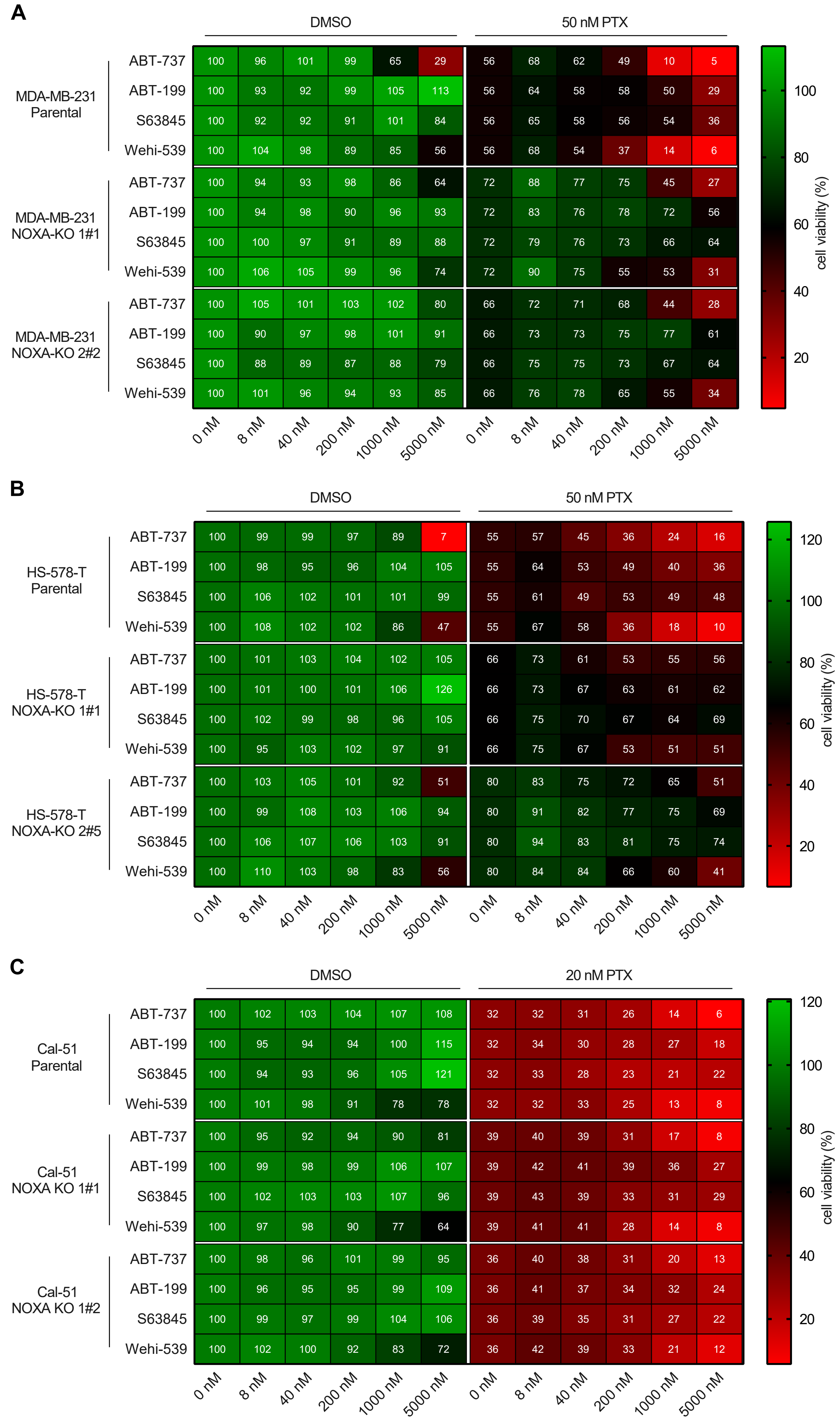
A
DMSO
50 nM PTX
MDA-MB-231 Parental
MDA-MB-231 NOXA-KO 1#1
MDA-MB-231 NOXA-KO 2#2
ABT-737 100 96 101 99 65 29 56 68 62 49 10 5
ABT-199 100 93 92 99 105 113 56 64 58 58 50 29
S63845 100 92 92 91 101 84 56 65 58 56 54 36
Wehi-539 100 104 98 89 85 56 56 68 54 37 14 6
ABT-737 100 94 93 98 86 64 72 88 77 75 45 27
ABT-199 100 94 98 90 96 93 72 83 76 78 72 56
S63845 100 100 97 91 89 88 72 79 76 73 66 64
Wehi-539 100 106 105 99 96 74 72 90 75 55 53 31
ABT-737 100 105 101 103 102 80 66 72 71 68 44 28
ABT-199 100 90 97 98 101 91 66 73 73 75 77 61
S63845 100 88 89 87 88 79 66 75 75 73 67 64
Wehi-539 100 101 96 94 93 85 66 76 78 65 55 34
0 nM
8 nM
40 nM
200 nM
1000 nM
5000 nM
0 nM
8 nM
40 nM
200 nM
1000 nM
5000 nM
100
80
60
40
20
cell viability (%)
B
DMSO
50 nM PTX
HS-578-T Parental
HS-578-T NOXA-KO 1#1
HS-578-T NOXA-KO 2#5
ABT-737 100 99 99 97 89 7 55 57 45 36 24 16
ABT-199 100 98 95 96 104 105 55 64 53 49 40 36
S63845 100 106 102 101 101 99 55 61 49 53 49 48
Wehi-539 100 108 102 102 86 47 55 67 58 36 18 10
ABT-737 100 101 103 104 102 105 66 73 61 53 55 56
ABT-199 100 101 100 101 106 126 66 73 67 63 61 62
S63845 100 102 99 98 96 105 66 75 70 67 64 69
Wehi-539 100 95 103 102 97 91 66 75 67 53 51 51
ABT-737 100 103 105 101 92 51 80 83 75 72 65 51
ABT-199 100 99 108 103 106 94 80 91 82 77 75 69
S63845 100 106 107 106 103 91 80 94 83 81 75 74
Wehi-539 100 110 103 98 83 56 80 84 84 66 60 41
0 nM
8 nM
40 nM
200 nM
1000 nM
5000 nM
0 nM
8 nM
40 nM
200 nM
1000 nM
5000 nM
120
100
80
60
40
20
cell viability (%)
C
DMSO
20 nM PTX
Cal-51 Parental
Cal-51 NOXA KO 1#1
Cal-51 NOXA KO 1#2
ABT-737 100 102 103 104 107 108 32 32 31 26 14 6
ABT-199 100 95 94 94 100 115 32 34 30 28 27 18
S63845 100 94 93 96 105 121 32 33 28 23 21 22
Wehi-539 100 101 98 91 78 78 32 32 33 25 13 8
ABT-737 100 95 92 94 90 81 39 40 39 31 17 8
ABT-199 100 99 98 99 106 107 39 42 41 39 36 27
S63845 100 102 103 103 107 96 39 43 39 33 31 29
Wehi-539 100 97 98 90 77 64 39 41 41 28 14 8
ABT-737 100 98 96 101 99 95 36 40 38 31 20 13
ABT-199 100 96 95 95 99 109 36 41 37 34 32 24
S63845 100 99 97 99 104 106 36 39 35 31 27 22
Wehi-539 100 102 100 92 83 72 36 42 39 33 21 12
0 nM
8 nM
40 nM
200 nM
1000 nM
5000 nM
0 nM
8 nM
40 nM
200 nM
1000 nM
5000 nM
120
100
80
60
40
20
cell viability (%)

Supp. Fig. 5

Gerlinde Karbon et al

A

	50nM PTX				
	DMSO	ABT-737	ABT-199	S63845	Wehi-539
MCF-7	2	2	1	1	2
T47D	5	2	3	12	1
ZR-75-1	7	7	7	5	9
SKBR3	5	1	2	2	1
MDA-MB-231	5	7	8	3	3
HS-578-T	4	2	3	6	4e-001
Cal-51	4	2	4	4	2
BT20	3	2	5	14	2

B

**MDA-MB-231**	DMSO						50 nM PTX					
	0 nM	8 nM	40 nM	200 nM	1000 nM	5000 nM	0 nM	8 nM	40 nM	200 nM	1000 nM	5000 nM
ABT-737	0	2	4	2	3	3	2	2	2	1	2	1
ABT-199	0	3	4	3	5	8	2	2	2	2	2	1
S63845	0	5	5	5	9	15	2	3	3	2	3	12
Wehi-539	0	3	3	3	2	2	2	2	3	3	1	1

C

**HS-578-T**	DMSO						50 nM PTX					
	0 nM	8 nM	40 nM	200 nM	1000 nM	5000 nM	0 nM	8 nM	40 nM	200 nM	1000 nM	5000 nM
ABT-737	0	2	2	2	2	6	5	5	4	6	3	2
ABT-199	0	5	5	8	6	9	5	5	3	5	5	8
S63845	0	5	6	6	5	6	5	3	5	5	6	6
Wehi-539	0	7	8	5	6	6	5	4	7	5	4	5

D

**Cal-51**	DMSO						20 nM PTX					
	0 nM	8 nM	40 nM	200 nM	1000 nM	5000 nM	0 nM	8 nM	40 nM	200 nM	1000 nM	5000 nM
ABT-737	0	4	6	6	5	6	6	6	6	6	8	7
ABT-199	0	4	6	6	7	8	6	6	6	7	7	7
S63845	0	4	4	5	4	7	6	4	3	3	3	3
Wehi-539	0	6	6	7	6	6	6	5	6	7	7	6

E

**BT20**	DMSO						10 nM PTX					
	0 nM	8 nM	40 nM	200 nM	1000 nM	5000 nM	0 nM	8 nM	40 nM	200 nM	1000 nM	5000 nM
ABT-737	0	5	6	5	3	4	2	7	8	8	9	6
ABT-199	0	7	5	5	5	4	2	6	6	6	7	3
S53845	0	6	6	8	10	14	18	13	7	9	9	8
Wehi-539	0	3	4	4	4	3	2	7	6	6	7	5

F

**MCF-7**	DMSO						10 nM PTX					
	0 nM	8 nM	40 nM	200 nM	1000 nM	5000 nM	0 nM	8 nM	40 nM	200 nM	1000 nM	5000 nM
ABT-737	0	7	9	9	5	7	7	13	12	8	9	5
ABT-199	0	7	7	6	4	9	7	10	9	8	7	3
S63845	0	2	3	4	2	1	7	5	4	4	1	2
Wehi-539	0	4	4	4	5	3	7	8	8	9	8	6

G

**T47D**	DMSO						10 nM PTX					
	0 nM	8 nM	40 nM	200 nM	1000 nM	5000 nM	0 nM	8 nM	40 nM	200 nM	1000 nM	5000 nM
ABT-737	0	3	6	5	4	5	4	6	6	6	5	6
ABT-199	0	4	3	5	3	5	4	6	5	4	3	3
S63845	0	5	5	4	5	5	4	3	5	4	4	6
Wehi-539	0	4	5	5	5	6	4	4	7	5	6	8

H

**ZR-75-1**	DMSO						50 nM PTX					
	0 nM	8 nM	40 nM	200 nM	1000 nM	5000 nM	0 nM	8 nM	40 nM	200 nM	1000 nM	5000 nM
ABT-737	0	4	4	6	5	8	8	8	4	4	4	3
ABT-199	0	7	4	4	4	9	8	5	5	4	4	6
S63845	0	6	8	6	8	6	5	5	5	6	5	3
Wehi-539	0	11	16	14	12	16	8	9	11	4	3	3

I

**SKBR3**	DMSO						10 nM PTX					
	0 nM	8 nM	40 nM	200 nM	1000 nM	5000 nM	0 nM	8 nM	40 nM	200 nM	1000 nM	5000 nM
ABT-737	0	3	4	5	7	7	4	5	6	4	5	5
ABT-199	0	3	4	6	5	6	4	3	5	6	6	5
S63845	0	12	11	4	2	3	7	15	5	3	1	1
Wehi-539	0	7	4	3	5	8	4	4	5	4	4	7

J

Cell line	Drug	DMSO 0 nM	DMSO 8 nM	DMSO 40 nM	DMSO 200 nM	DMSO 1000 nM	DMSO 5000 nM	50 nM PTX 0 nM	50 nM PTX 8 nM	50 nM PTX 40 nM	50 nM PTX 200 nM	50 nM PTX 1000 nM	50 nM PTX 5000 nM
MDA-MB-231 Parental	ABT-737	0	2	3	8	7	5	1	3	2	8	2	1
	ABT-199	0	5	6	7	7	11	1	3	2	4	2	2
	S63845	0	5	5	5	9	15	1	3	3	2	3	12
	Wehi-539	0	7	5	7	9	9	1	5	4	11	5	5e-001
MDA-MB-231 NOXA-KO 1#1	ABT-737	0	4	6	5	10	6	6	9	7	7	4	1
	ABT-199	0	7	5	16	11	13	6	7	7	9	6	4
	S63845	0	8	7	13	10	12	6	5	5	5	10	4
	Wehi-539	0	9	9	9	7	7	6	5	4	4	9	4
MDA-MB-231 NOXA-KO 2#2	ABT-737	0	7	3	2	3	6	9	11	12	10	9	6
	ABT-199	0	2	1	5	3	4	9	11	12	13	11	10
	S63845	0	4	3	2	3	9	9	13	11	11	8	7
	Wehi-539	0	2	3	3	3	1	9	12	11	11	14	7

K

Cell line	Drug	DMSO 0 nM	DMSO 8 nM	DMSO 40 nM	DMSO 200 nM	DMSO 1000 nM	DMSO 5000 nM	50 nM PTX 0 nM	50 nM PTX 8 nM	50 nM PTX 40 nM	50 nM PTX 200 nM	50 nM PTX 1000 nM	50 nM PTX 5000 nM
HS-578-T Parental	ABT-737	0	6	6	6	8	2	5	9	8	7	5	5
	ABT-199	0	6	5	5	6	2	5	7	6	5	6	7
	S63845	0	12	11	9	8	8	5	8	10	6	6	5
	Wehi-539	0	12	11	11	10	16	5	11	9	8	3	2
HS-578-T NOXA-KO 1#1	ABT-737	0	4	6	4	6	8	8	8	8	9	9	11
	ABT-199	0	9	4	2	1	10	8	9	7	6	12	15
	S63845	0	5	5	4	6	5	8	3	7	8	7	10
	Wehi-539	0	10	7	5	6	9	8	14	12	12	14	15
HS-578-T NOXA-KO 2#5	ABT-737	0	6	2	3	2	13	5	9	9	6	5	4
	ABT-199	0	4	2	3	7	13	5	6	4	4	6	5
	S63845	0	3	1	1	3e-001	2	5	5	5	4	5	2
	Wehi-539	0	4	7	4	4	6	5	7	10	5	3	3

L

Cell line	Drug	DMSO 0 nM	DMSO 8 nM	DMSO 40 nM	DMSO 200 nM	DMSO 1000 nM	DMSO 5000 nM	20 nM PTX 0 nM	20 nM PTX 8 nM	20 nM PTX 40 nM	20 nM PTX 200 nM	20 nM PTX 1000 nM	20 nM PTX 5000 nM
Cal-51 Parental	ABT-737	0	3	3	4	3	8	2	3	4	3	2	1
	ABT-199	0	1	2	3	2	7	2	3	3	3	2	2
	S63845	0	3	2	3	5	9	2	3	3	2	2	3
	Wehi-539	0	4	3	3	1	1	2	2	3	3	2	1
Cal-51 NOXA KO 1#1	ABT-737	0	1	2	3	4	4	2	3	2	2	2	1
	ABT-199	0	2	2	3	3	6	2	3	2	2	3	3
	S63845	0	3	4	4	5	12	2	4	3	3	3	4
	Wehi-539	0	4	4	5	6	4	2	4	4	3	2	3
Cal-51 NOXA KO 1#2	ABT-737	0	5	4	4	5	4	2	3	3	3	2	2
	ABT-199	0	3	3	3	6	6	2	3	2	3	3	2
	S63845	0	3	4	3	3	4	2	3	2	3	2	6
	Wehi-539	0	4	2	3	6	5	2	3	2	3	3	3

Supp. Fig 6

Gerlinde Karbon et al

A

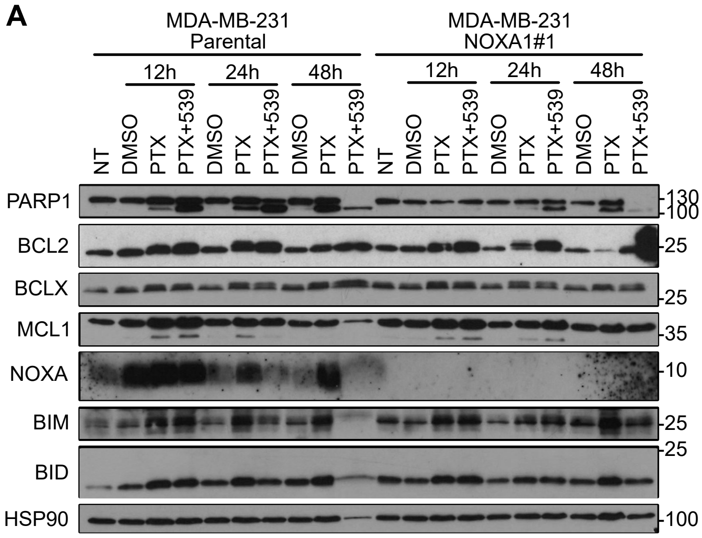

Supplement: Supplementary file 1 — Suppl. Figs. 1-6 [file 41419_2021_4415_MOESM1_ESM.pdf]
