# Supplementary material for: The BH3-only protein NOXA serves as an independent predictor of breast cancer patient survival and defines susceptibility to microtubule targeting agents
Source: Cell Death Dis. 2021 Dec 13;12(12):1151. doi: 10.1038/s41419-021-04415-y (PMC8668920; doi:10.1038/s41419-021-04415-y)
Supplement: Supplementary file 3 — Suppl. legends [file 41419_2021_4415_MOESM3_ESM.docx]

# Supplementary Figure Legend

***Supplementary Figure 1: Pearson correlation analysis of BCL2 family members. (A)*** *mRNA expression in 92 BC tissues was compared* *explore co-expression patterns. (****B)*** *Six randomly selected patient samples with mRNA levels below or above the mean expression value were selected and analyzed by western blotting. 40µg of protein lysate were loaded per lane.*

***Supplementary Figure 2: Assessment of metabolic activity with MTT assay after treatment with BH3-mimetics and paclitaxel****. (****A-D****)* *The* *indicated cell lines were treated with 10 or 50 nM paclitaxel alone or in combination with graded concentrations of different BH3‑mimetics (ABT-737, ABT-199, S63845 and Wehi-539) for 48h. DMSO was used as a control. Heatmap is showing the mean value of metabolic activity ranging from 0 to 100% assessed by MTT-assay. (****A****) MCF-7 (n=4), (****B****) T47D (n=4), (****C****) ZR-75-1 (n=4), (****D****) SKBR3 (n=4).*

***Supplementary Figure 3: Paclitaxel increases Sensitivity towards BH3-mimetics.*** *(****A-H****)* *The* *indicated cell lines were treated with graded doses of paclitaxel in combination with 1 µM of BH3-mimetics for 48 h. Data are shown as mean ±SEM. *: p-value <0.05, **: p‑value <0.01,***: p-value <0.001. (****A****) MDA-MB-231(n=4), (****B****) HS-578-T (n=4), (****C****) Cal-51 (n=4), (****D****) BT-20 (n=5), (****E****) MCF-7 (n=4), (****F****) T47D (n=3), (****G****) ZR-75-1 (n=4), (****H****) SKBR3 (n=3).*

***Supplementary Figure 4: NOXA deletion protects from PTX and BH3-mimetic induced apoptosis.*** *Parental and two NOXA-KO clones of each TNBC cell line were treated with increasing doses of indicated BH3-mimetics alone (DMSO) or in combination with 20 or 50 nM PTX for 48h. Heatmap is showing the mean value of metabolic activity assessed with MTT-assay. (****A****) MDA-MB-231 (n=5), (****B****) HS-578-T (n=4), (****C****) Cal-51 (n=5).*

***Supplementary Figure 5: SEM for the heatmaps shown in Fig. 4 (A-E), Suppl. Fig. 2 (F-I) and Fig. 6 (J-L).***

***Supplementary Figure 6: NOXA-deficiency reduces PTX killing in MDA-MB-231 cells.*** Asynchronous cells of the indicated genotypes were treated with solvent (DMSO), PTX (50 nM) or PTX (50 nM) + Wehi-539 (1 µM) for up to 48h. Cell lysates were subjected to SDS-page and western blot analyses.

***Supplementary Table 1:*** Association of mRNA expression of BCL2 family members with clinicopathological characteristics of 92 breast cancer patients diagnosed and treated at the Medical University of Innsbruck, AT. Abbreviations used: LN, lymph node status; MP, menopausal status; HER2, human epidermal growth factor receptor 2 status; ER, oestrogen receptor status; n.a, not available. p-values were calculated using non-parametric Mann–Whitney.
