# Supplementary material for: The BH3-only protein NOXA serves as an independent predictor of breast cancer patient survival and defines susceptibility to microtubule targeting agents
Source: Cell Death Dis. 2021 Dec 13;12(12):1151. doi: 10.1038/s41419-021-04415-y (PMC8668920; doi:10.1038/s41419-021-04415-y)
Supplement: Supplementary file 4 — Suppl. materials [file 41419_2021_4415_MOESM4_ESM.docx]

# Supplementary Material

Table 1 | Media composition for BC cell lines.

| Cell line | Media |
| --- | --- |
| MCF-7 | MEM +Earle (Lonza, BE12-125F)  +10% FCS (FBS, PAA laboratories, A15-151)  +2 mM L- Glutamin (Sigma G7513)  +100 U/ml penicillin and 100 μg/ml streptomycin (Lonza 17-602E)  +Non-Essential AA 1x (Gibco 11140-035)  +0.01 mg/ml bovine Insulin (sigma-Aldrich I1882) |
| SKBR3 | Mc Coy`s 5A Medium (Sigma Aldrich BE12-688F)  +10% FCS (FBS, PAA laboratories, A15-151)  +2 mM L- Glutamin (Sigma G7513)  +100 U/ml penicillin and 100 μg/ml streptomycin (Lonza 17-602E) |
| T47D | RPMI 1640 (Sigma Aldrich, R0883)  +10% FCS (FBS, PAA laboratories, A15-151)  +2 mM L- Glutamin (PAA laboratories, M11-004)  +100 U/ml penicillin and 100 μg/ml streptomycin (Lonza 17-602E) |
| BT20  MDA-MB-231  HS-578-T | EMEM + Earle (Lonza, BE12-125F)  +10% FCS (FBS, PAA laboratories, A15-151)  +2 mM L- Glutamin (Sigma G7513)  +100 U/ml penicillin and 100 μg/ml streptomycin (Lonza 17-602E)  +Non-Essential AA 1x (Gibco 11140-035) |
| ZR-75-1 | RPMI 1640 (Sigma Aldrich, R0883)  +10% FCS (FBS, PAA laboratories, A15-151)  +2 mM L- Glutamin (Sigma G7513)  +100 U/ml penicillin and 100 μg/ml streptomycin (Lonza 17-602E)  +4,5 g/l Glucose (Sigma-Aldrich D83752) |
| Cal-51 | DMEM (Sigma Aldrich, D5671)  +10% FCS (FBS, PAA laboratories, A15-151)  +2 mM L-Glutamin (Sigma G7513)  +100 U/ml penicillin and 100 μg/ml streptomycin (Lonza 17-602E) |

Table 2 | List of Primary antibodies for Western blot.

| Primary Antibodies | | Source (dilution) |
| --- | --- | --- |
| Rabbit | **MCL1** | Santa Cruz 819 (1:1000) |
| Mouse | **NOXA** | Calbiochem, clone 114C307 (1:400) |
| Rat | **BCLW** | Clone 13F9 (Wehi/Alexis) (1:500) |
| Mouse | **BCL2** | Clone S100, gift from Andreas Srasser (1 µg/ml) |
| Rabbit | **BCLX** | Cell Singanlling 2764 (1:1000) |
| Rabbit | **BIM** | Enzo Life Sciences ADI-AAP-330-E (1:500) |
| Rabbit | **PUMA** | CS 4976 (1:500) |
| Rabbit | **BAD** | CS 9292 (1:500) |
| Rabbit | **BAX** | CS 2772 (1:1000) |
| Rabbit | **BAK** | CS 3814 (1:1000) |
| Rabbit | **BCLB** | CS 3869 (1:500) |
| Rat | **BID** | Clone 8C3 (1:1000) |
| Rabbit | **BOK** | Clone 1-5-4, gift from Thomas Kaufmann, Bern (1:500) |
| Rabbit | **PARP1** | Cell Signalling 9542 (1:1000) |
| Mouse | **CDC27** | Clone Bd610455 (1:500) |
| Mouse | **Cyclin B** | Clone V152 (1:1000) |
| Rabbit | **GAPDH** | Cell Signalling 2118, clone 14C10 (1:5000) |
| Mouse | **HSP90** | Santa Cruz 13119, clone F8 (1:1000) |

**Table 3 | List of Primers.** Primers and Probes for BCLB and BAK were determined with the Primer Express software 2.0 (Applied Biosystems, Thermo Scientific, CA, USA). Primers and probe for *BCL2* family members were purchased from Thermo Fisher Scientific (Waltham, Massachusetts, MA, USA, Thermo Fisher Scientific).

| Gene | Primer/Genbank |
| --- | --- |
| Mycoplasma test | Forward: GGG AGC AAA CAG GAT TAG ATA CCC T  Reverse: TGC ACC ATC TGT CAC TCT GTT AAC CTC |
| BCL2L10 (BCLB; isoform 1) | NM_001306168.1  Forward: TCTCCAAAAAGCCAGTGGAAA  Reverse: GGAAGCGCCCACGAGG  TQM: AAGAAGTGACAAAAGCCATCCGTGCCA |
| BCL2L10 (BCLB; isoform 2) | NM_020396.4  Forward: TCTCCAAAAAGCCAGTGGAAA  Reverse: CGCGCCTGGCTGCA  TQM: AGAAGTGACAAAAGCCATCCCAGCCG |
| BAK1 | NM_001188.4  Forward: CGGGTTGGGCCAGGAT  Reverse: AAGCCATTTTTCAGGTCTCAGTG  TQM: CCGGCAGGCTGATCCCGTCC |
| Gene | **Assay IDs** |
| PMAIP1 (NOXA): | Hs00560402_m1 |
| BCL2: | Hs00608023_m1 |
| BCL2L1 (BCLX): | Hs00236329_m1 |
| MCL1: | Hs01050896_m1; |
| BCL2L2 (BCL-W): | Hs00187848_m1 |
| PUMA: | Hs00560402_m1 |
| BCL2L11 (BIM): | Hs01076940_m1 |
| BAX: | Hs00180269_m1 |
| BOK: | Hs00261296_m1 |
| BID: | Hs00609632_m1 |
|  |  |
| Gene | **Guide RNA sequence for generation of NOXA KO cells** |
| sgNoxa1 | TCGAGTGTGCTACTCAACTC AGG |
| sgNoxa2 | ACGCTCAACCGAGCCCCGCG CGG |

Table 4 | List of Chemical Compounds

| Compound | Company |
| --- | --- |
| ABT-737 | Selleck Biochem S1002 |
| ABT-199 | Apexbio A8194 |
| S63845 | Selleck Chemicals, 333 S8383 |
| Wehi‑539. | Apexbio A3935 |
| Paclitaxel | Sigma-Aldrich, T7191 |
